# Potential of Probiotic Frozen Blackcurrant Products: Consumer Preference, Physicochemical Characterization, and Cell Viability

**DOI:** 10.3390/foods10040792

**Published:** 2021-04-07

**Authors:** Kati Väkeväinen, Noora Rinkinen, Roosa-Maria Willman, Jenni Lappi, Kaisa Raninen, Anna Kårlund, Santtu Mikkonen, Carme Plumed-Ferrer, Marjukka Kolehmainen

**Affiliations:** 1 Institute of Public Health and Clinical Nutrition, University of Eastern Finland, P.O. Box 1627, 70210 Kuopio, Finland; rinkinennoora@gmail.com (N.R.); roosa-maria.willman@uef.fi (R.-M.W.); kaisa.raninen@uef.fi (K.R.); anna.karlund@uef.fi (A.K.); marjukka.kolehmainen@uef.fi (M.K.); 2 Faculty of Business, Tourism and Hospitality, Savonia University of Applied Sciences, P.O. Box 6, 70201 Kuopio, Finland; jenni.lappi@savonia.fi; 3 SIB Labs, University of Eastern Finland, P.O. Box 1627, 70210 Kuopio, Finland; 4 Department of Applied Physics, University of Eastern Finland, P.O. Box 1627, 70210 Kuopio, Finland; santtu.mikkonen@uef.fi; 5 Department of Environmental and Biological Sciences, University of Eastern Finland, P.O. Box 1627, 70210 Kuopio, Finland; 6 Probitat Ltd., Microkatu 1R, 70210 Kuopio, Finland; carme.plumed@probitat.fi

**Keywords:** blackcurrant berries, vegan, sensory evaluation, probiotic, *Lactoplantibacillus plantarum*

## Abstract

Blackcurrant is a healthy, affordable, and traditionally gardened berry that, thus far, has been underused in food applications. From the consumers’ point of view, the acidic taste of blackcurrants is a challenge; therefore, these berries have mainly been utilized for sugary juice production. This research study aimed to develop a frozen vegan blackcurrant product with pleasant sensory properties and potential probiotic function. A candidate probiotic, *Lactoplantibacillus plantarum* Q823, was used in the manufacturing process. The physicochemical properties, nutritional composition, and consumer preference for the developed product were assessed, as was the viability of *L. plantarum* Q823 during storage time and in an in vitro gastrointestinal model. Consumers (*n* = 71) perceived the developed product to be pleasant. *L. plantarum* Q823 had high viability counts (log colony forming units (cfu) g^−1^ 7.0 ± 0.38) in the final product, although the viability of *L. plantarum* Q823 during storage time needs to be enhanced to obtain a probiotic product. Thus, within an optimized formulation, blackcurrant berries represent a potential raw material for functional frozen food products.

## 1. Introduction

The consumer interest for novel, health-promoting vegan foods is markedly increasing [1,2], together with preferences for natural, low processed products [3]. There is also a growing demand for foods suitable for specific diets, such as gluten-free and lactose-free products [4,5]. The food industry requires products with a long storage time, and consumers prefer foods that are convenient [6]. Within the category of frozen snack products, healthier dairy products have been developed by adding fruits or berries (e.g., References [7,8]), but more studies on the development of vegan frozen products are needed [9].

Berries are part of the healthy Nordic diet [10]; in the Finnish diet, they constitute the number one source of polyphenols [11]. Berries and their polyphenolic compounds, such as anthocyanins, have positive health effects on postprandial glucose metabolism [12,13,14], oxidative stress [15], and inflammation [16].

Blackcurrant (*Ribes nigrum*) berries are rich in anthocyanins [17]. The plant is feasible and economical to cultivate, making blackcurrant berries a promising ingredient for healthy products. In Finland, black and green currants (990 tons) were the second most produced berries after strawberries in 2018 [18]. However, blackcurrant berries are underused in the food industry and they are processed mainly as jams and juices containing a substantial amount of sugar. The key barriers to the wider utilization of blackcurrant berries are their sour taste and astringent mouthfeel, which consumers may perceive as being unpleasant without the addition of sweeteners [19,20].

Thermal processes used to manufacture many berry products change the aroma, color, texture, and health-promoting properties of the final products [21]. Among other phenolic compounds, anthocyanins are degraded during thermal processes of berries, leading to losses of color and possible health effects [22]. Thus, novel food technology solutions are needed to produce blackcurrant products that secure high nutritional value and sensory quality.

Lactic acid bacteria (LAB) are traditionally used in fermented foods for their versatile technological properties and their ability to prolong the storage time of foods [23], and they have enormous potential for the development of new functional foods. The viability of LAB in aerated frozen products is challenged through high redox value, oxygen, whipping and freezing, and acidity [24]. Lactobacilli are resistant to oxygen [25], so they can survive under different manufacturing conditions. Therefore, they are extensively used in the food industry and are studied as starter cultures and probiotics [26].

A probiotic product should include a minimum of log colony forming units (cfu) g^−1^ 6–7 viable cells per 100 g of food [27]. Prebiotics, such as dietary fibers and oligosaccharides [9,28] have been used to enhance the viability of probiotics in foods. However, there are only few studies on the utilization of fruits and berries [8] as prebiotic ingredients, taking advantage of their prebiotic potential, flavor characteristics, and healthiness.

Our aim was to utilize scientific know-how in nutrition, food technology, biotechnology, and consumer research to develop a nutritious and pleasant-tasting, potentially probiotic frozen snack product from blackcurrants utilizing LAB-fermented quinoa. In practice, the physicochemical properties and consumer liking of three different flavor variants were analyzed. The viability of *Lactoplantibacillus plantarum* Q823 (formerly known as *Lactobacillus plantarum*) was studied during 90-day storage, and an in vitro gastrointestinal digestion model was used to assess the probiotic functionality of the three product variants.

## 2. Materials and Methods

### 2.1. Bacterial Strains and Culture Conditions

A candidate probiotic, *L. plantarum* Q823, previously isolated from quinoa [29] and proven to survive in acidic products [30,31], was used in the manufacturing process of blackcurrant products. *L. plantarum* Q823 was stored at −80 °C, grown in De Man, Rogosa and Sharpe (MRS) broth (Lab M, Bury, Lancashire, UK), and incubated at 30 °C for 16 h prior to inoculation.

### 2.2. Manufacturing Process

Prior to manufacturing the blackcurrant products for further investigation, their composition and flavoring were optimized by a trained in-house-panel (*n* = 5, 27–50 years) using a ranking test [32]. The first prototype contained blackcurrants (54.9%), fermented quinoa base (11.0%, of which 12% was quinoa), sugar (18.3%), rapeseed oil, alpha-cyclodextrin (Wacker Chemie AG, Munich, Germany), locust bean gum (Unipektin Ingredients AG), and guar gum. Consequently, a blackcurrant product that was only flavored with sugar was further developed. Sugar was used in the product to mask the acidic flavor of the blackcurrants and increase the liking of the product. Moreover, product variants were tested, flavored with sugar and one of the following options: cola, lemon, pepper, peppermint, spruce shoot, vanilla, or a combination of vanilla and lemon.

**Figure 1:**
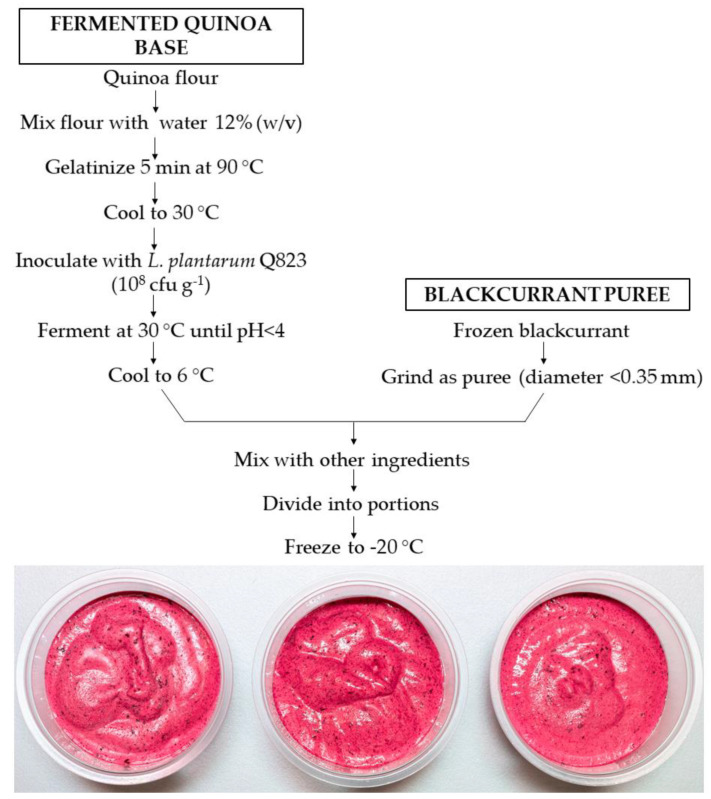
The manufacturing process of frozen blackcurrant products.

As an outcome of the ranking tests, three blackcurrant products were manufactured: (1) a sugar-only flavored product (SBP), (2) a product flavored with sugar and vanilla (VBP), and (3) a product flavored with sugar, vanilla, and lemon (VLBP). Vanilla and lemon flavoring were applied to observe whether additional flavoring increases the palatability of blackcurrant products. The manufacturing process included the preparation of smooth blackcurrant puree and fermented quinoa base (Figure 1).

Frozen blackcurrants (*Ribes nigrum* [Öjebyn]) of Finnish origin were provided by Pakkasmarja Ltd. (Suonenjoki, Finland). Whole frozen blackcurrants were mashed using an industrial colloid mill (PVS Systemtechnik GmbH, blade diameter <0.35 mm), and the obtained puree was stored at −20 °C until the product was manufactured. The blackcurrant puree was not heat-treated during the manufacturing process to maintain its natural properties. The quinoa flour was mixed with water (12% *w/v*) and pasteurized for 5 min at 90 °C (60 rpm). The mixture was rapidly cooled to 30 °C and inoculated by log cfu mL^−1^ 8 of *L. plantarum* Q823 (1% *v/v*) [31]. The quinoa base was fermented at 30 °C overnight to reach pH < 4 and stored at 6 °C until the product was manufactured.

An 8 kg batch of each product was manufactured in a pilot plant (SavoGrow Ltd., Suonenjoki, Finland). SBP contained blackcurrants (50.1%), fermented quinoa base (17.4%, of which 12% was quinoa), sugar (16.7%), water, rapeseed oil, alpha-cyclodextrin (Wacker Chemie AG, Munich, Germany), locust bean gum (Unipektin Ingredients AG), and guar gum. The VBP variant also included vanilla flavoring (0.1% *w/w*), and the VLBP also included vanilla flavoring (0.1% *w/w*) and lemon juice (2% *w/w*). All the ingredients were mixed in a stainless-steel dish, whipped to obtain an airy structure, divided in plastic dishes, and frozen to −20 °C.

### 2.3. Physicochemical Analysis

The pH, total titratable acidity (TTA), viscosity, melting rate, overrun, and nutritional composition of the SBP, VBP, and VLBP samples were determined in triplicate at 20 °C (*n* = 3). pH, TTA, and viscosity (PCR Instruments, Rotary Viscometer PCE-RVI3, Model 20, spindle 7, Hampshire/Southampton, UK) were measured according to Väkeväinen et al. [31]. To discard excess air, the samples were melted to 20 °C and mixed with a spatula for 30 s prior to obtaining the measurements. The melting rate was determined by placing 100 g of the samples onto a stainless-steel mesh (4 holes per cm^2^) over a dish and weighting the amount (g) of the sample that drained into the dish over a period of 120 min [33]. The overrun was calculated during the manufacturing process using the following formula: Overrun (%) = (weight of the ingredients prior to whipping − weight of the product)/(weight of the product) × 100 [28].

The fat, protein, ash (total solids), moisture, and total dietary fiber contents were determined according to Association of Official Analytical Collaboration (AOAC) International 2005 [34] standard methods. Petroleum ether was used as the solvent for the fat analysis. The total carbohydrate content was calculated by subtracting the percentage sum of the moisture, protein, fat, and ash from 100%. The total fiber content for SBP, VBP, and VLBP was calculated from the analyzed fiber content in the blackcurrant puree (AOAC 985.29 combined with AOAC 991.43), and the specification-based calculated values from the other ingredients.

### 2.4. Consumer Sensory Evaluation

Sensory evaluation of SBP, VBP, and VLBP was performed using a consumer panel. The evaluations were performed in the sensory evaluation laboratories [35] of the University of Eastern Finland and Savonia University of Applied Sciences. Sensory evaluation was conducted according to the ethical principles of the University of Eastern Finland. All subjects gave their informed consent for inclusion before they participated in the study.

The non-trained consumers were recruited from the Kuopio area (Finland) by distributing printed and electronic versions of the research call in local academies, grocery shops, and gyms. The exclusion criteria were: pregnancy, breastfeeding, daily smoking, celiac disease, or allergies to berries, citrus fruits, nuts, and cereals. A total of 71 consumers (60 females and 10 males, 1 other gender), aged 18–64 years, participated in the sensory evaluation (Table A1). Sensory evaluation sessions were designed, and data were collected with EyeQuestion software (Elst, The Netherlands, version 4.11.27). Prior to the consumer sensory evaluation, the microbiological safety of SBP, VBP, and VLBP was ensured by plating total mesophiles (37 °C, 24 h, Plate Count Agar, LabM, Lancashire, UK), yeasts and molds (30 °C, 48 h, Sabouraud Agar supplemented with chloramphenicol, LabM), and coliforms (37 °C, 24 h, Violet Red Bile Agar, LabM). Only products containing no coliforms were accepted for the sensory evaluation.

The sensory evaluation of the samples was conducted after they had been stored for 1 week at −20 °C, according to the guidelines found in Lawless and Heymann [36]. Prior to the evaluation, the samples (40 g) were taken to room temperature (20 °C) for 20 min, and they were then served to the consumers in transparent plastic cups that were covered with a lid. All the samples were coded with random three-digit-numbers and presented to the consumers in a randomized order. The consumers were asked to rinse their mouth with distilled water prior to tasting each sample. The evaluated properties were selected based on the terms obtained during the optimization of product formulation and the literature [8,37,38]. The sensory evaluation protocol was validated with a trained in-house panel (*n* = 5). The consumers were asked to evaluate the overall liking, sweetness, sourness, berryness, and texture of the samples using a nine-point hedonic scale, ranging from 1 = I do not like at all, to 9 = I like very much [36]. At the end of the sensory evaluation, the consumers were asked to rank all the samples according to their overall preference, ranging from 1 = I liked the most, to 3 = I liked the least. The consumers were also asked whether they perceived the samples to be sorbets, snacks, ice creams, frozen desserts, or other products. In the end, the consumers had the opportunity to provide voluntary written comments regarding the evaluated samples.

### 2.5. Viable Cell Counts during the 90-Day Storage

The viability of *L. plantarum* Q823 in the SBP, VBP, and VLBP samples was determined in triplicate during 90-day storage at the following time points: 1, 7, 14, 21, 30, 60, and 90 days [9]. The LAB viability counts were enumerated on MRS agar (30 °C, 48 h), and the results were calculated as log cfu g^−1^.

### 2.6. In Vitro Survival of L. plantarum Q823

In the SBP sample, the survival of *L. plantarum* Q823 in the gastrointestinal tract was evaluated using a modified INFOGEST static in vitro simulation of gastrointestinal food digestion [39]. The digestion simulation was performed in triplicate at storage time points of 1, 14, and 21 days. To simulate the oral phase, 75 g of the sample was diluted 1:1 in sterile water and homogenized for 60 s (Stomacher 400, Seward Medical, London, UK). In the gastric phase, the sample was diluted 1:1 with pepsin (from porcine gastric mucosa, final concentration 2000 U mL^−1^, Sigma-Aldrich, St. Louis, MO, USA) and the pH was adjusted to 2.0 (1 M HCl). The sample was incubated for 2 h at 37 °C (115 rpm). The sample was further diluted 1:1 with 0.2 M NaHCO_2_ (Sigma-Aldrich). To simulate the conditions in the small intestine, pancreatin (final concentration of 2.0 mg mL^−1^, ≥1000 U mL^−1^, MP Biomedicals, Solon, OH, USA), and bile salts (final concentration 8.2 mg mL^−1^, Ox-bile, Sigma-Aldrich) were added, and the pH was set at 7.0 (1 M NaHCO_3_, Sigma-Aldrich). The sample was incubated for 2 h at 37 °C (140 rpm). Samples for microbiological analysis were gathered after each digestion phase. The LAB viability counts were enumerated as described in Section 2.5. To identify the enumerated colonies as *L. plantarum*, DNA extraction (Nucleo^®^ Tissue Kit, Macherey-Nagel, Düren, Germany) was performed for single colonies (*n* = 16) randomly picked from the MRS agar and *L. plantarum* species-specific polymerase chain reaction (PCR) with primers planF (5′-CCG TTT ATG CGG AAC ACC TA-3′), and planpenR (5′-TCG GGA TTA CCA AAC ATC AC-3′) [40] was applied to obtain fragments of approximately 318 bp. *L. plantarum* Q823 was used as the positive control.

### 2.7. Statistical Analysis

All the results were expressed as mean ± standard deviation (SD). Analysis of variance (ANOVA) and Tukey’s test (*p* < 0.05) were used for the physicochemical and microbiological data (IBM SPSS Statistics, Version 27, Armonk, NY, USA). The sensory evaluation data were analyzed with IBM SPSS Statistics and EyeOpenR (EyeQuestion Version 4.11.63, EyeOpenR Data Analysis, EyeQuestion Software, Elst, The Netherlands and Qi Statistic Ltd., Kings Hill, UK). Since the overall liking scores did not follow a normal distribution (Shapiro–Wilk test), the Friedman test (*p* < 0.05) was used to analyze the statistically significant differences among the three samples and the Wilcoxon test was used for pairwise comparisons. The Principal Component Analysis (PCA, ref. [41]) was applied for studying the dependencies between the study variables. Additionally, stepwise regression was applied for finding the variables affecting most to the overall liking measured with consumer sensory evaluation.

## 3. Results

### 3.1. Physicochemical Analysis

The obtained pH and TTA values of the SBP, VBP, and VLBP samples were similar (Table 1). The lowest pH and highest TTA values were obtained for VLBP. The viscosity of the SBP, VBP, and VLBP samples did not differ significantly (76.5 ± 3.00) (Tukey’s test, *p* > 0.05) (Table 1). No dripping of the sample through the stainless-steel mesh was observed when the melting rate was tested for 120 min. VBP had the highest overrun value (137.9% ± 6.4%) (Table 1). The protein content of the blackcurrant products was 0.8 ± 0.04 g 100 g^−1^, the fat content was 6.8 ± 0.00 g 100 g^−1^, the carbohydrate content was 30.5 ± 0.42 g 100 g^−1^, and the total fiber content was 7.2 g 100 g^−1^ (Table 1).

The obtained pH for the three blackcurrant products was similar to vegan passionfruit ice creams (pH 3.0–3.3, [9]) and slightly lower than the pH in typical dairy-based probiotic frozen berry products (Table 1) (pH 5.3–4.5, [8]).

The melting properties of the blackcurrant products differed notably from probiotic butiá ice cream [37] and vegan passionfruit ice cream [9], which completely melted during 60–145 min. The melting properties can be influenced by the overrun, the emulsifying properties of the ingredients, and the concentrations of lipids and proteins [42]. For SBP, VBP, and VLBP, alpha-cyclodextrin is the most probable cause for the lack of structural changes during melting.

Generally, the overrun of dairy ice creams is 80–100% [24]. In our study, the overrun values were higher than those previously reported with frozen vegan fruit products (28.8–56.6% [9]) and berry products (33.2–40.7% [43]).

The nutritional content of SBP, VBP, and VLBP was similar since the variants only differed in their flavoring (Table 1). The blackcurrant content of the SBP, VBP, and VLBP samples was 50.1%, which is greater than the content of this berry in previously developed vegan frozen products (17.8–18.8% [9]) or in dairy-based products, where it ranges from 5–15% [7] to 49% [44]. The total fat content on SBP, VBP, and VLBP was only 6.8 ± 0.00 g 100 g^−1^. Furthermore, the products contained 7.2 g 100 g^−1^ fiber, demonstrating their promising nutritional quality. It is important to note that the reported fat content of SBP, VBP, and VLBP was obtained by combining database-derived fat content for the blackcurrant puree, fermented quinoa base, and rapeseed oil [45]. The fat content based on the database was used due to the fat-binding properties of alpha-cyclodextrin.

### 3.2. Consumer Sensory Evaluation

The overall consumer liking of SBP, VBP, and VLBP was rated as 7.5 ± 1.27 on a hedonic 1–9 scale (Table 2). No statistically significant differences were observed in the overall liking, sweetness, sourness, berryness, or texture of the samples (Friedman, *p* > 0.05). At the end of the sensory evaluation, the consumers ranked the samples based on their overall preference. With this test, SBP was the product sample that the consumers most often liked (Friedman, *p* < 0.05) and VBP was the sample they liked the least. The obtained results highlight that sugar as flavoring is sufficient to obtain a pleasant product and mask the acidic flavor of blackcurrants. Hence, adding vanilla or lemon as a flavoring did not further increase the consumers’ preference for the product. The most chosen product categories were sorbets (33.8%), frozen desserts (31.0%), and snack products (21.1%) (Table A1). The consumers stated that the most important factors they consider when buying a product are: (1) the price (67.6%), (2) healthiness (66.2%), and (3) origin (66.2%).

**Figure A1:**
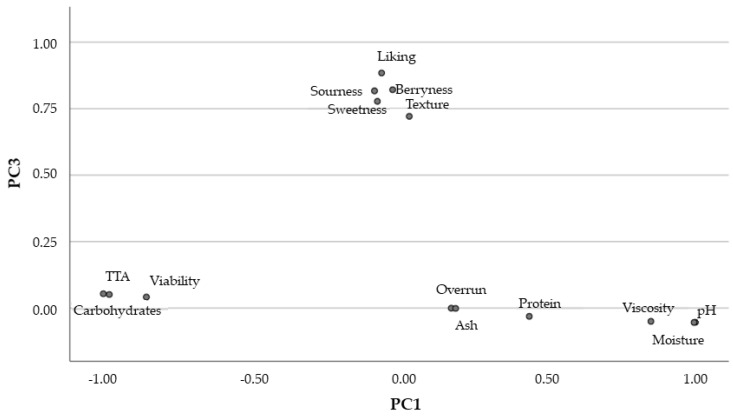
Loadings of the Principal components 1 and 3 given by the Principal Component Analysis (PCA).

The PCA assigned the study variables in three components (Table 3). The PCA with three components explained a total of 87.8% of the observed variance, and component-wise percentages are shown in Table 3. First and second components included physicochemical and viability properties of the products, and the third one included the results from the consumer sensory evaluation. Figure A1 illustrates how the component loadings separate the variables, specifically the variables closely related to overall liking. The PCA showed that the liking indications from consumer evaluation did not have significant dependency on the physicochemical properties. 

This was confirmed with regression analysis, done in a stepwise manner, which showed that the most important predictors for overall liking were the other properties studied with the consumer sensory evaluation. Due to limitations, set by collinearity, only one of the physicochemical variables could be included in the regression model at a time. The tests with the regression model indicated that the most important property affecting overall liking, additional to the consumer sensory evaluation, was the protein content of the products (Table A2).

### 3.3. Viable Cell Counts during the 90-Day Storage

**Figure 2:**
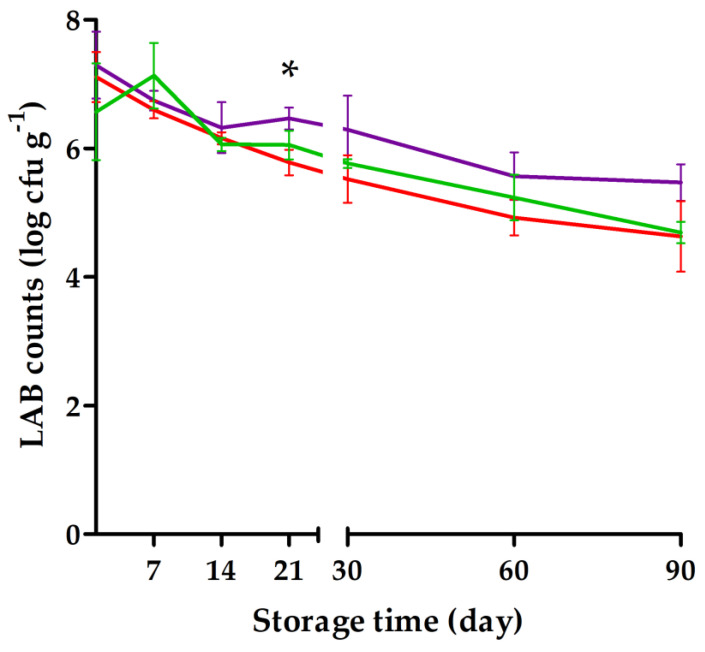
The viable counts of lactic acid bacteria (log colony forming units (cfu) g^−1^, average ± SD) of the frozen blackcurrant products during 90-day storage at −20 °C. Sugar-only flavored blackcurrant product (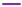), product flavored with sugar and vanilla (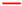), and product flavored with sugar, vanilla, and lemon (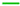). The sugar-only flavored blackcurrant product was significantly different from that of the product flavored with sugar and vanilla at an individual time point: * *p* < 0.05 (Tukey test). Results are means of triplicates.

The LAB viability counts after day 1 of storage were log cfu g^−1^ 7.0 ± 0.38 (Figure 2). The counts remained above log cfu g^−1^ 6 at the following storage time points: day 7 (log cfu g^−1^ 6.9 ± 0.27), day 14 (log cfu g^−1^ 6.2 ± 0.13), and day 21 (log cfu g^−1^ 6.1 ± 0.34).

### 3.4. In Vitro Survival of L. plantarum Q823

**Figure 3:**
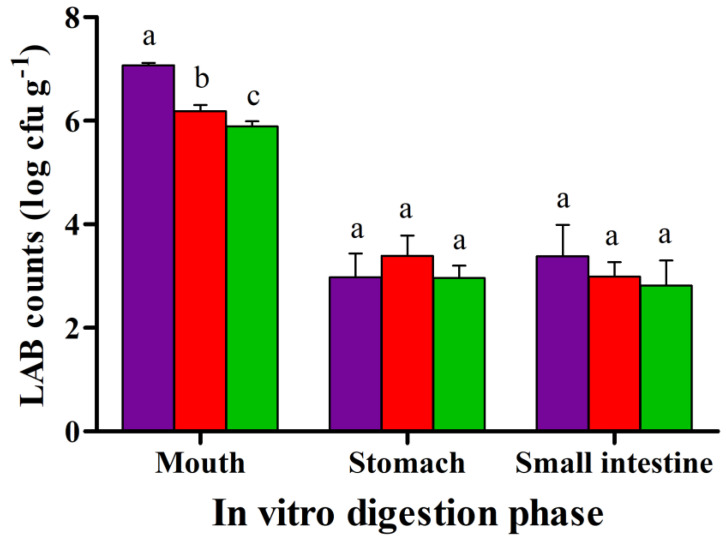
The viable counts of lactic acid bacteria (log cfu g^−1^, average ± SD) in sugar-only flavored blackcurrant product (SBP) in an in vitro model after 1 (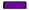), 14 (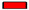), and 21 (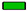) days of storage at −20 °C. The viable counts with different letters are significantly different according to Tukey’s test (*p* < 0.05). Results are means of triplicates.

At day 1 of storage, the viability counts of *L. plantarum* Q823 were log cfu g^−1^ 7.1 ± 0.04. The values notably decreased after the gastric phase (Figure 3); in contrast, the oral and small intestine phases did not have significant effects on the survival of *L. plantarum* Q823. The notable decrease in the LAB viability counts in the gastric phase (Figure 3) was likely due to the very low pH (2.0) used in this study. The INFOGEST model [39] recommends that the pH in the gastric phase should be <3.0 for viability studies. However, there are no clear standards on the exact pH, and values of 2.1–2.6. have been used [9,46].

## 4. Discussion

The aim of this study was to utilize scientific know-how in nutrition, food technology, biotechnology, and consumer research to develop a nutritious and pleasant-tasting, potentially probiotic frozen snack product from blackcurrants utilizing LAB-fermented quinoa. The optimized blackcurrant products developed in the current study were successful in overcoming the challenges regarding sour taste and astringent mouthfeel [19,20], and the LAB viability counts remained ≥log cfu g^−1^ 6 for 21 days of storage (Figure 2). Thus, sugar-sweetened SBP, VBP, and VLBP represent good vehicles for the delivery of anthocyanins and other bioactive or prebiotic constituents, as well as probiotic bacteria, all potentially supporting human well-being.

The high overall liking scores of the SBP, VBP, and VLBP samples are in line with the results reported in previous research (Table 2) with blueberry [43] and *Myrtus communis* products [8], thus demonstrating their potential as pleasant frozen snack products.

Previously, the neutral pH of frozen dairy products has been found to increase consumer acceptance and to support the survival of probiotics [24]. However, in line with the present results, the low pH of dairy-based butiá ice cream [37] and vegan passionfruit ice creams did not decrease their palatability [9]. Additionally, *L. plantarum* Q823 has previously been shown to tolerate a pH 3.4–4.2 in food matrices [30,31], and the survival of probiotics under low pH in dairy-based and vegan ice creams has been demonstrated [9,37]. The results obtained from our in vitro model showed that *L. plantarum* Q823 has the potential to survive through gastric and small intestine digestion. Exposing the bacteria to an in vitro gastric environment at pH below 3.0 rather well predicts the probiotic survival in the physiological conditions in vivo [39].

However, the high overrun values (Table 1) of the blackcurrant products—expressing the amount of air that is incorporated into a frozen product during the manufacturing process [24]—may have decreased the LAB viability counts [47] during storage. Although lactobacilli are resistant to oxygen, increasing the overrun has been suggested to also expose the bacteria to physical stress by mechanical mixing, which may affect viability [47]. The products retained the minimum probiotic viability count (i.e., log cfu g^−1^ 6–7) recommended for products with a daily consumption of 100 g [27]. The obtained counts for the 21-day storage would be sufficient for artisanal manufacture [48], but longer storage viability is needed for industrial scale manufacturing. In future product refinements, the viability counts of *L. plantarum* Q823 can easily be increased by inoculating a higher density of *L. plantarum* Q823 during the manufacturing process or by adding microencapsulated microbes to the final product [49].

The high overrun values (Table 1) suggested that the blackcurrant products have a light and pleasant texture. Furthermore, since consumers find fast-melting products unpleasant to consume [50], the ability of SBP, VBP, and VLBP to retain their shape and airy texture throughout the observed 120 min period is a desired result.

The lack of recently developed vegan, berry-based probiotic frozen products makes it challenging to evaluate the nutritional quality of the SBP, VBP, and VLBP samples. However, the protein content of a frozen product containing berries has previously been reported as 0.6–0.7 g 100 g^−1^, the fat content as 11.2–14.4 g 100 g^−1^, and the carbohydrate content as 21.9–28.6 g 100 g^−1^ [9]. The fat content of SBP, VBP, and VLBP was lower than with other similar products. In addition, it is important to notice that rapeseed oil added to SBP, VBP, and VLBP contributed to the high proportion of unsaturated fat in the blackcurrant products of the present study.

Previously, the anthocyanins of a similar frozen blackcurrant product have been determined at the time of consumption [14]. However, the anthocyanin content of berry products has been shown to decrease during 6 months of storage [22]. Hence, in the future product refinements, the stability of anthocyanins in the frozen blackcurrant products should be verified.

## 5. Conclusions

To the best of our knowledge, this is the first study to evaluate functional frozen blackcurrant products. The consumers (*n* = 71) perceived the developed flavor variants to be pleasant and the nutritional quality of the products was seen as promising, although this maybe challenging due to strong astringency and even sourness of the blackcurrant. The incorporation of candidate probiotic *L. plantarum* Q823 gives the product functional properties. However, the viability of *L. plantarum* Q823 needs to be enhanced in future product refinements to fulfill the requirements for probiotic products throughout the storage time.

## Figures and Tables

**Table 1:** pH, TTA (mL of NaOH 10 g^−1^), viscosity (Pas), overrun (%), and nutritional composition (g 100 g^−1^, fresh matter) (average ± standard deviation (SD)) of frozen blackcurrant products.

Product	SBP	VBP	VLBP
pH	3.09 ± 0.01 ^a^	3.07 ± 0.01 ^ab^	3.03 ± 0.03 ^b^
TTA (mL of NaOH)	25.5 ± 0.87 ^a^	25.6 ± 0.21 ^ab^	27.0 ± 0.21 ^b^
Viscosity (Pas)	79.9 ± 3.59 ^a^	75.4 ± 5.43 ^a^	74.2 ± 3.45 ^a^
Overrun (%)	116.9 ± 1.54 ^a^	137.9 ± 6.4 ^b^	118.4 ± 0.75 ^a^
Moisture	61.9 ± 0.04 ^a^	61.6 ± 0.03 ^c^	61.0 ± 0.01 ^b^
Ash	0.35 ± 0.05 ^a^	0.37 ± 0.02 ^a^	0.35 ± 0.02 ^a^
Protein	0.78 ± 0.04 ^a^	0.74 ± 0.00 ^a^	0.75 ± 0.07 ^a^
Fat ^1^	6.8 ^a^	6.8 ^a^	6.8 ^a^
Carbohydrates ^2^	30.1 ± 0.06 ^a^	30.4 ± 0.03 ^c^	31.1 ± 0.05 ^b^
Total fiber ^3^	7.2	7.2	7.2

Products with different letters are significantly different according to Tukey’s test (*p* < 0.05). Results are means of triplicates. SBP = Sugar-only flavored blackcurrant product, VBP = product flavored with sugar and vanilla, and VLBP = product flavored with sugar, vanilla, and lemon. ^1^ Calculated based on content of fat in the ingredients used. ^2^ Calculated by subtracting the percentage sum of moisture, protein, fat, and ash from 100%. ^3^ Calculated based on the analysis in blackcurrant puree and calculated content of fiber in the other ingredients.

**Table 2:** Consumer (*n* = 71) liking scores (average ± SD) of frozen blackcurrant products. 1 = I do not like at all, 9 = I like very much.

	SBP	VBP	VLBP
Overall liking	7.2 ± 1.45	7.6 ± 1.19	7.6 ± 1.11
Sweetness	7.3 ± 1.61	7.4 ± 1.58	7.7 ± 1.17
Sourness	6.9 ± 1.83	7.2 ± 1.63	7.4 ± 1.48
Berryness	7.7 ± 1.46	7.5 ± 1.56	7.8 ± 1.23
Texture	7.2 ± 1.79	7.3 ± 1.63	7.3 ± 1.56

No statistically significant differences were observed within products according to Friedman’s test (*p* < 0.05). SBP = Sugar-only flavored blackcurrant product, VBP = product flavored with sugar and vanilla, and VLBP = product flavored with sugar, vanilla, and lemon.

**Table 3:** Rotated component matrix from the Principal Component Analysis (PCA) and the percentage of variance explained by each component. Loadings < 0.2 are hidden from the table.

		Component	
	1	2	3
Explained variance %	40.5	24.0	23.3
Carbohydrates	−0.998		
Moisture	0.998		
pH	0.994		
TTA	−0.978	0.200	
Viability	−0.853	0.520	
Viscosity	0.848	0.527	
Overrun		−0.984	
Ash		−0.982	
Protein	0.438	0.898	
Liking			0.884
Berryness			0.821
Sourness			0.817
Sweetness			0.777
Texture			0.721

## Data Availability

Data available on request. The data presented in this study are available on request from the corresponding author. The authors do not have permission to share the data.

## Appendix A

**Table A1:** Background information of the consumers (*n* = 71).

Background Information	*n* = 71	%
**Gender**		
Male	10	14.1
Female	60	84.5
Other	1	1.4
**Age**		
18–24	22	31.0
25–30	14	19.7
31–40	14	19.7
41–50	8	11.3
51–65	13	18.3
**Job description**		
Working	38	53.5
Student	31	43.7
Other	2	2.8
**Consumption of berries**		
Daily	19	26.8
Few times a week	25	35.2
Once a week	9	12.7
Few times a month	16	19.7
Rarely	2	2.8
Never	0	0
**Do you look at a product’s nutritional information before buying it?**		
Yes	14	19.7
Yes, sometimes	52	73.2
No	5	7.0
**Most important choosing factors when buying a product ^1^**		
Package	2	2.7
Healthiness	47	66.2
Habit	21	29.6
Price	48	67.6
Organic or local product	7	9.9
Origin	47	66.2
Brand	1	1.4
Ecological aspect/Sustainability	13	18.3
Quality	24	33.8
Other	1	1.4

^1^ Consumers could choose several options.

## Appendix C

Table A2 shows the evolution of stepwise regression model for overall liking. The stepwise process included five steps, numbered in the table as Models 1–5. The variables were added to the model in the order of prediction ability, i.e., the strongest predictor was inserted first, then the second strongest, etc. From the final model, Model 5, even though the protein was inserted to model before berryness, the absolute value of the standardized coefficient is lower and thus protein is the weakest of the predictors.

**Table A2:** Model evolution for stepwise regression.

		Unstandardized Coefficients		Standardized Coefficients		
Model	Variables in Model	β	Standard Error	β	t	Significance
1	(Constant)	3.302	0.343		9.632	0.000
	Sweetness	0.563	0.045	0.652	12.476	0.000
2	(Constant)	1.839	0.319		5.758	0.000
	Sweetness	0.414	0.040	0.479	10.307	0.000
	Texture	0.354	0.036	0.462	9.937	0.000
3	(Constant)	1.578	0.305		5.173	0.000
	Sweetness	0.291	0.045	0.336	6.504	0.000
	Texture	0.309	0.035	0.403	8.914	0.000
	Sourness	0.210	0.040	0.275	5.232	0.000
4	(Constant)	7.168	2.543		2.819	0.005
	Sweetness	0.292	0.044	0.338	6.601	0.000
	Texture	0.312	0.034	0.407	9.062	0.000
	Sourness	0.201	0.040	0.264	5.037	0.000
	Protein	−7.360	3.324	−0.090	−2.214	0.028
5	(Constant)	7.696	2.521		3.053	0.003
	Sweetness	0.274	0.044	0.317	6.169	0.000
	Texture	0.282	0.036	0.367	7.801	0.000
	Sourness	0.153	0.044	0.201	3.490	0.001
	Protein	−8.405	3.311	−0.103	−2.539	0.012
	Berryness	0.126	0.051	0.141	2.483	0.014
